# Functional vision tests as clinical trial outcome measures in ophthalmology: a scoping review

**DOI:** 10.1136/bmjopen-2024-097970

**Published:** 2025-05-27

**Authors:** Shabnam Raji, Arun James Thirunavukarasu, Laura Jayne Taylor, Robert Edward MacLaren

**Affiliations:** 1 Nuffield Laboratory of Ophthalmology, Department of Clinical Neurosciences, University of Oxford, Oxford, UK; 2 Oxford Eye Hospital, Oxford University Hospitals NHS Foundation Trust, Oxford, UK; 3 Oxford University Clinical Academic Graduate School, University of Oxford, Oxford, UK

**Keywords:** OPHTHALMOLOGY, Clinical Trial, GENETICS

## Abstract

**Abstract:**

**Objectives:**

To identify currently available functional vision tests and evaluate their use as clinical trial outcome measures in ophthalmology.

**Design:**

Scoping review using the PRISMA-ScR (Preferred Reporting Items for Systematic Reviews and Meta-analysis Extension for Scoping Reviews) guidelines.

**Methods:**

A literature search was conducted in MEDLINE and Embase (via Ovid) for articles published between 1 January 2003 and 1 August 2024. Additional grey literature was sourced from institutional repositories, conference proceedings and a manual citation search. Article screening was conducted against a predefined inclusion criteria by two independent, masked reviewers, with a third reviewer acting as arbiter. The inclusion criteria were English language articles which feature a test assessing functional vision in patients with an ophthalmological disease. Details of source characteristics, test methodology and accessibility and evidence of test validation were collected.

**Results:**

Of 2665 articles returned by the search, 73 were included and 45 unique tests of functional vision were identified. Diseases affecting the peripheral retina were mainly affected, accounting for 77% (56 out of 73) of the diseases featured in all included studies. Overall, 82% (37 out of 45) functional vision tests reported evidence of statistical validation with varying robustness. Functional vision tests were mapped to domains of orientation and mobility, facial recognition, observer-rated task performance, visual search and driving. Obstacle courses assess vision-guided orientation and mobility, correlate highly with clinical measures of visual function in severe peripheral retinal disease and have been validated for use in clinical trials. Their requirement of physical space and time limits utility in multicentre trials; equivalent tests leveraging virtual reality and eye tracking technologies are in development. Early iterations of visual search tests to simulated realistic scenes have demonstrated discriminative ability, even in paediatric patients.

**Conclusions:**

Functional vision tests can facilitate research into future novel ophthalmological treatments that prioritise patients in terms of how clinical benefit is defined. The principal barriers to the uptake of these tests are lack of accessibility, low quality validation and that many tests remain early in their development stage. This review captures the current landscape of functional vision tests and serves as a reference for investigators and regulatory bodies to evaluate the suitability of these tests for ophthalmic clinical trials.

**STRENGTHS AND LIMITATIONS OF THIS STUDY**

- This review provides the first evaluation of functional vision tests in ophthalmology, focusing on their potential as clinical trial outcome measures.
- A comprehensive grey literature search was performed to minimise the risk of bias.
- Due to heterogeneity in reported test validation, in-depth statistical analysis of validation data was not undertaken.
- Incomplete or insufficiently detailed data in the included studies limited the scope of the analysis.

## Introduction

 Functional vision tests measure how well individuals can interact with their visual environment,^1^ and these tests may characterise certain eye diseases better than standard clinical measures of visual function and patient-reported outcome measures (PROMs).^2^ Functional vision is distinct from visual function which describes the physiological function of the eye and associated visual system, often through contrived clinical tests such as perimetry or visual acuity. Functional vision tests are based on activities of daily living in several domains: mobility, object identification, facial recognition and reading, among others. They output objective scores and can conflate aspects of visual acuity, spatial vision, cognition, colour vision, light sensitivity and adaptation to assess overall function.^3^ They also consist of relatively complex tasks that assess higher-order visual processing, which may offer a more holistic understanding of visual impairment. In this way, they are highly pertinent measures of a patient’s overall quality of life and have broad potential application as clinically meaningful outcome measures in ophthalmology clinical trials.

Currently accepted visual function outcome measures in ophthalmology include best-corrected visual acuity, perimetry, full-field stimulus testing, microperimetry and mobility testing.^4 5^ Despite standardisation, visual acuity remains a gross characterisation of overall vision, insensitive to changes in retinal function away from the fovea and displays poor reliability in patients with visual impairment.^6^ Standard automated perimetry has been the gold standard for detecting optic nerve damage and has been used effectively as an outcome measure in glaucoma trials.^7^ However, perimetry is limited by low test–retest reliability, particularly in those with poor steady, central fixation in macular disease and certain oculomotor abnormalities, such as nystagmus.^6^ Fundus-controlled perimetry, or microperimetry, has gained favour in this regard and has become a key endpoint in several clinical trials.^8^

Structural outcome measures in ophthalmology can offer precise, highly reproducible assessments of disease progression and can delineate anatomical biomarkers. However, these measures may not be applicable if structure and function do not reliably correlate, for instance, where there is amblyopia or a gene defect affecting enzymes of the visual cycle. In these cases, it is unclear how anatomical changes in the eye translate to patient benefit.^6^

In other medical specialties, functional tests have already been established as key clinical trial endpoints, such as in stroke medicine and multiple sclerosis.^9 10^ The US Food and Drug Administration has published specific guidelines on patient-centred drug development^11^ to prioritise the impact of novel treatments on patients. Similarly, the WHO’s International Classification of Functioning, Disability and Health framework classifies health in terms of functioning and disability in daily life.^12^ It provides the basis for a more integrated understanding of health, with emphasis on practical function rather than solely biomedical variables. Research is ongoing in ophthalmology clinical trials to align with this framework.

Here, a review was undertaken to identify currently available functional vision tests and evaluate their application as clinical trial outcome measures in ophthalmology.

## Methods

### Search strategy

A scoping review was selected due to the heterogeneity of articles found in the preliminary literature search, and to allow for more exploratory analysis of functional vision tests as an outcome measure. The review was undertaken in accordance with the PRISMA-ScR (Preferred Reporting Items for Systematic Reviews and Meta-Analyses extension for Scoping Reviews).^13^ A literature search was conducted in MEDLINE and Embase (both via Ovid). Publication dates were restricted from 1 January 2003 to 1 August 2024. A grey literature search was conducted to minimise publication bias and maximise the scope of the review. Grey literature sources included a manual citation search, Google Scholar, conference proceedings and the British Library Electronic Theses Online Service. The full Boolean search string with combined index and free text terms is detailed in online supplemental table S1.

Duplicates were manually removed by two reviewers. Title and abstract screening, and full text screening were conducted against a predefined inclusion criteria by two independent, masked reviewers, with a third reviewer acting as arbiter to resolve disagreement by casting a deciding vote.

### Inclusion and exclusion criteria

The inclusion criteria were as follows: (1) Written in the English language; (2) Is a primary research article; (3) Is not a retracted article; (4) Features a test designed for human patients; (5) Test assesses functional vision. Included tests were restricted to those used in patients with an ophthalmological disease. Psychophysical, visual function tests and PROMs were excluded. Although an important domain of functional vision, reading tests were excluded in this search as they have been subject to extensive literature review.^14^

### Data extraction and analysis

Key features of the included texts were charted by two independent, masked reviewers with results synthesised by one reviewer. Data on study design, patient characteristics, test methodology, visual function correlates, validity and repeatability evidence and accessibility were extracted. Specifically, articles were searched for evidence of the following: test responsiveness, inter-rater and intrarater reliability, test–retest reliability, content, construct and criterion validity. Repeatability and validity data were abstracted to only include statistical values of significance and correlation; purely qualitative statements were excluded. Data visualisation was performed with Microsoft Excel 2024 (Microsoft Corporation, USA) and Inkscape (V.0.92).

### Patient and public involvement

There was no direct patient or public involvement in this review.

## Results

**Figure 1:**
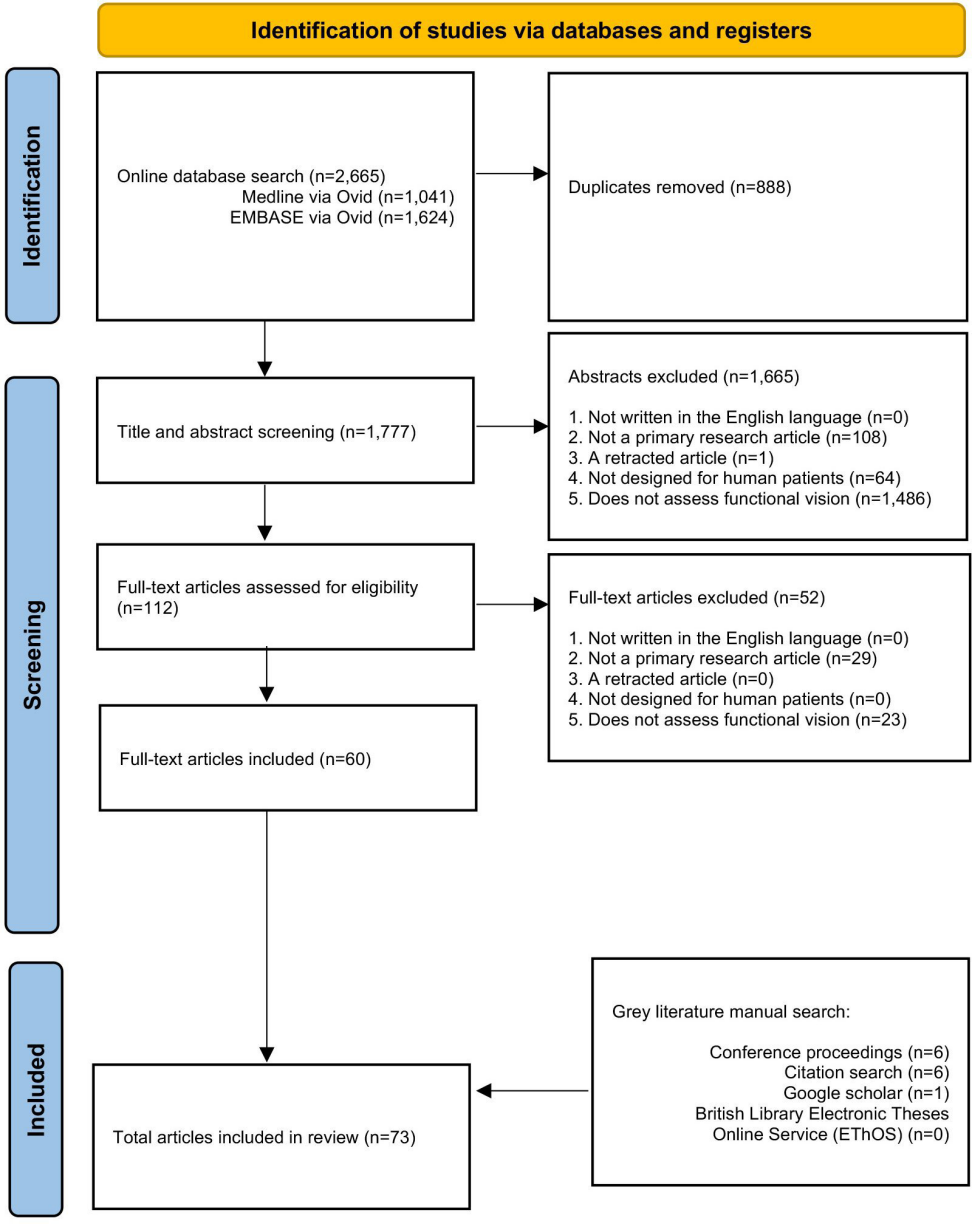
Preferred Reporting Items for Systematic Reviews and Meta-Analyses extension for Scoping Reviews (PRISMA-ScR) flow diagram of the study selection process.

**Figure 2:**
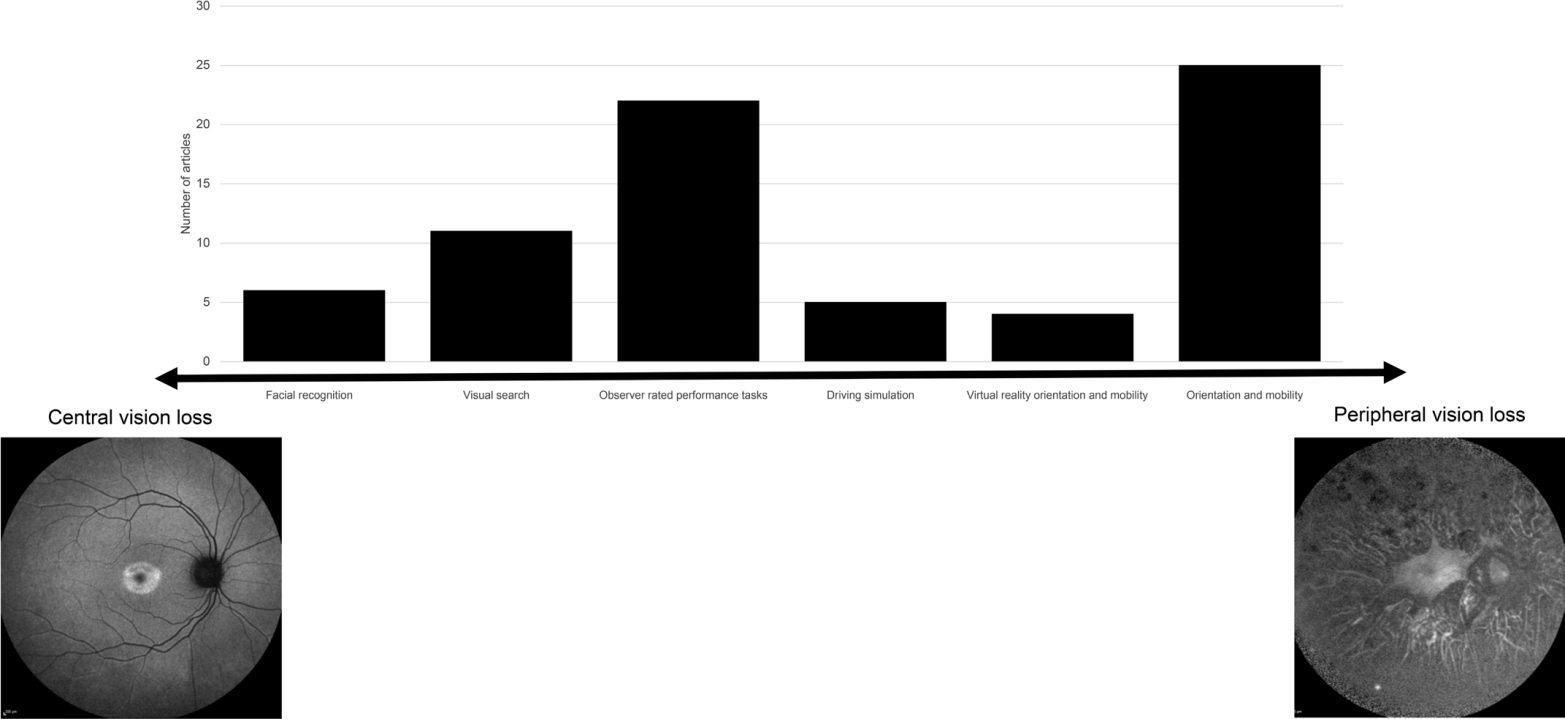
Number of included articles (n=73) contributing to each category of functional vision test. Six categories of functional vision test ordered on a continuum based on reported ability to measure central or peripheral vision loss. Exemplar fundus autofluorescence images depicting severe peripheral retinal degeneration due to *RPE65*-associated Leber’s congenital amaurosis (left) and discrete central atrophy within the macula due to *RPGR*-associated cone dystrophy (right). In some severe retinal degenerations, such as end-stage Leber’s congenital amaurosis, extensive peripheral degeneration encroaches centrally, leading to complete loss of light perception.

**Figure 3:**
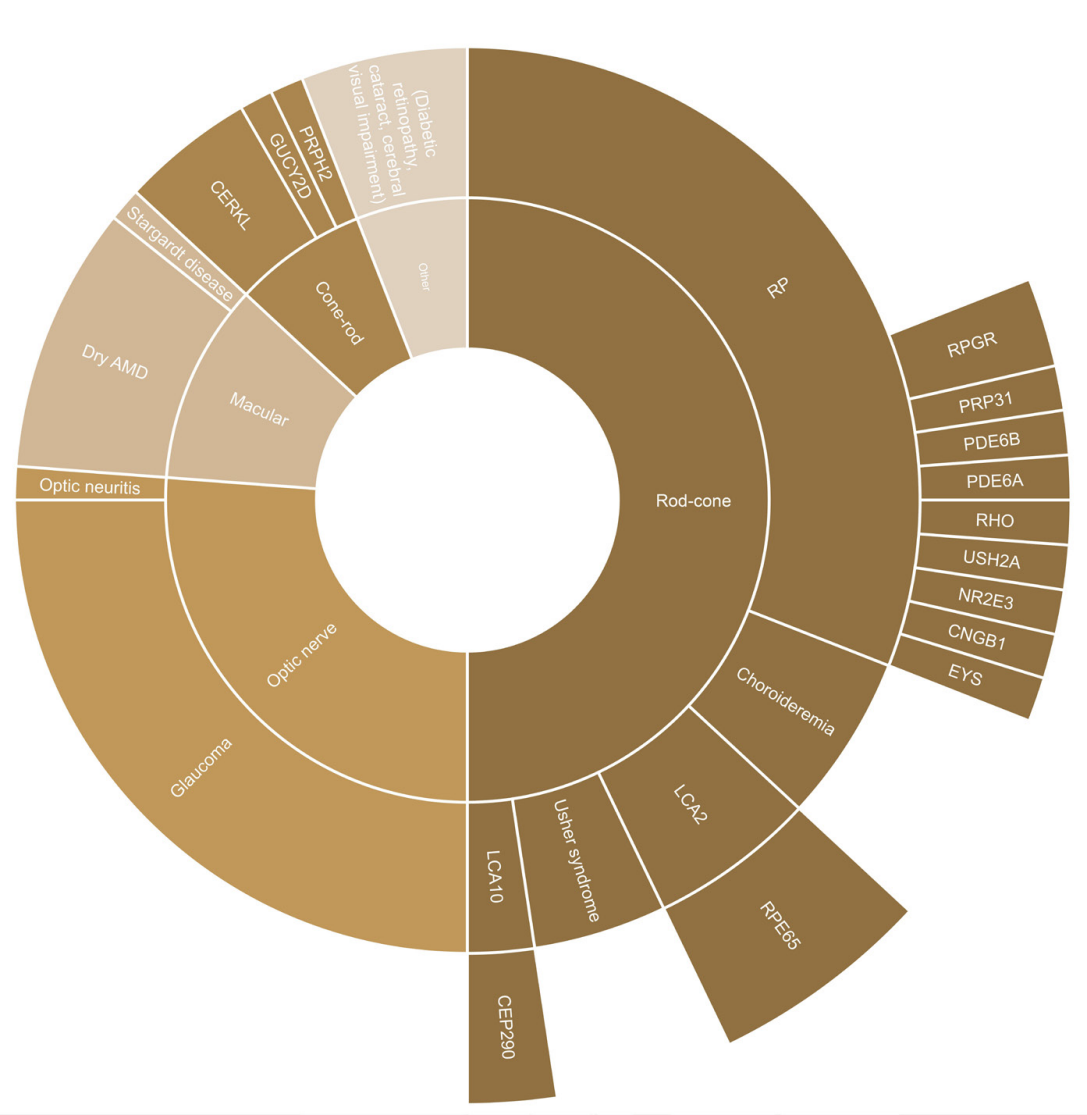
Disease of patient population in included articles (n=73) categorised by the structure of the eye affected, clinical phenotype and, where reported, genotype. AMD, age-related macular degeneration; RP, retinitis pigmentosa.

**Figure 4:**
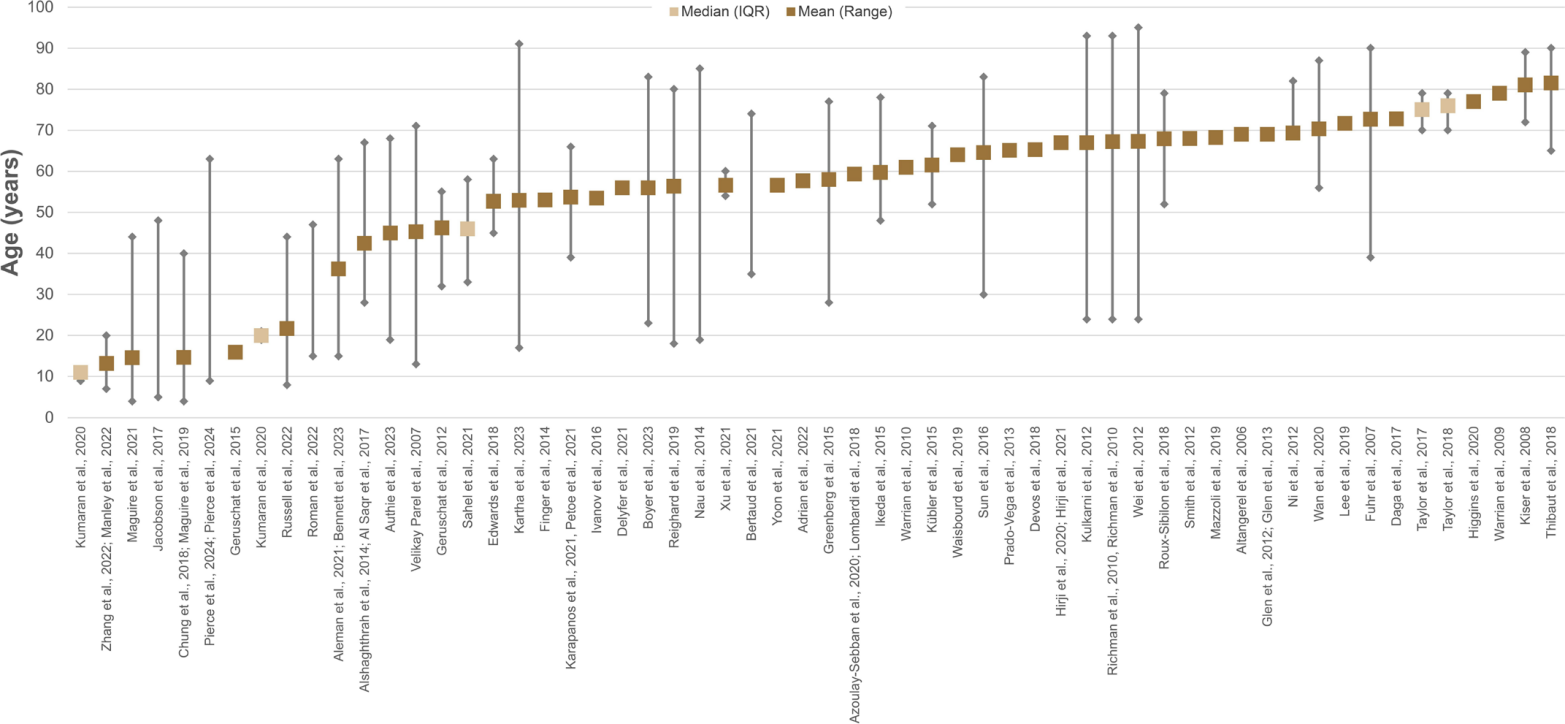
Reported age of the patient population assessed with functional vision tests. The dashed line demarcates age 18, below which signifies paediatric testing. Five articles were omitted as no age data was available. Note that there are few studies testing paediatric patient populations and even fewer suitable for preschool age children.

The initial search yielded 2665 articles. After screening, a total of 73 texts were included: 67 peer-reviewed publications and 6 conference abstracts. The full search and screening process is shown in figure 1. Source characteristics of all included studies are summarised in table 1. 45 unique functional vision tests were identified and listed in online supplemental table S2. An abridged list of functional vision tests is listed in table 2. All functional vision tests were grouped into thematic categories for further analysis and are illustrated in figure 2 along a continuum based on their reported ability to measure central or peripheral vision loss. The number of included articles contributing to each category of functional vision test is also shown in figure 2. Orientation and mobility and observer-rated performance tasks accounted for the highest number of articles found with 25 and 22, respectively. Virtual reality (VR) was the least represented with four articles, although all were published within the last 5 years which predicts an expanding area of research, in line with the growth of new technologies. Figure 3 illustrates the disease of the patient population in the included articles categorised by structure of the eye affected, clinical phenotype and genotype. Functional vision tests were mainly investigated in diseases affecting the peripheral retina, which accounted for 77% (56 out of 73) of the diseases featured in all included studies. Rod-cone dystrophies and optic nerve diseases were common, appearing in 37 and 19 articles, respectively. Cone-rod dystrophies and macular disease (both inherited and acquired) featured in fewer studies; 6 and 9, respectively. The number of patients within studies ranged from 4 to 192 and the distribution of reported patient age across all studies is displayed in figure 4. Only 14 out of 73 articles included a paediatric cohort of patient.

**Table 1:** Summary source characteristics of all included studies

Publication year	Number of studies
2005–2010	8
2011–2015	15
2016–2020	24
2021–2024	26
Study design	
Interventional study	
Phase I/II randomised controlled trial	3
Phase III randomised controlled trial	1
Pilot/feasibility	1
Observational studies	
Cross-sectional	49
Case series	10
Case-control	2
Cohort	1
Conference proceedings	
Abstract	6
Country of institutional affiliation	
North America	38
Europe	24
Asia	4
Oceania	4
Middle East	2
South America	1
Africa	0

**Table 2:** Patient population, reference standard, test outcomes and repeatability and validity data of all included studies featuring a functional vision test

Citation	Patient population	Functional vision test	Reference standard(s)	Test outcome(s)	Reported repeatability and validity data
Orientation and mobility (O&M)					
Roman *et al*, 2022^61^	10 patients with *GUCY2D-*associated and *CEP290-*associated Leber’s congenital amaurosis	Mobility test for rod-mediated vision	VA; FST	Navigation success over a fixed number of trials; Travel duration	Content validity—Mobility demonstrated a linear relationship with FST. No correlation between VA and mobilityConstruct validity—No significant difference between controls and patients in suprathreshold transit time (p=0.63). At threshold and dimmer luminance levels, transit times increased for both patients and normal subjects.
Sahel *et al*, 2021^15^	25 patients with retinitis pigmentosa and *RPE65-*associated Leber’s congenital amaurosis	StreetLab mobility course	VA; VF; CS; Dark adaptation	Course completion time; PWS; PPWS; Number of collisions; Walking initiation time; trajectory analyses/segments; Distance travelled	Construct validity—Patients performed worse than controls for PWS, PPWS, number of collisions and walking initiation time under both low and high illumination.
Bertaud *et al*, 2021	22 patients with glaucoma				Construct validity—No difference in mobility performance between patients and controls under photopic luminance. Under glare conditions, PWS and PPWS were significantly lower in patients than controls (p=0.049 and p=0.038, respectively). Mobility time was significantly longer in patients than controls (p=0.046). Distance travelled, mobility incidents and trajectory segmentations not significantly different between patients and controls.
Chung *et al*, 2018;^55^ Maguire *et al*, 2019^20^	19 patients with *RPE65-*associated Leber’s congenital amaurosis	Multi-Luminance Mobility Test (MLMT)	VA; VF; FST (white light)	MLMT binocular change score (number of collisions and time to navigate course)	Content validity—Variable correlation of accuracy score with quality-of-life questionnaire (r=−0.54 to −0.7). Correlation of mean accuracy score with VA ranged from 0.75 to 0.86. Correlation between mean accuracy score and total degrees of visual field ranged from −0.37 to −0.53.Construct validity—Able to distinguish controls from patients.Repeatability—High inter-grader agreement for scoring (Cohen’s kappa=97.9%). High concordance between scores at baseline visits ranging from 86% to 98%.Sensitivity to change—Over 1-year observation period, controls had an MLMT change score of 0, representing no change, and 20 patients had an MLMT change score of 0. Few patients had an MLMT change score of −1 or −2 (ie, a worsening).
Lam *et al*, 2024**^26^	18 patients with *NR2E3 and RHO-*associated retinitis pigmentosa			MLMT monocular change score	Construct validity—six out of seven *RHO* patients had stable or improved MLMT scores, including two patients that demonstrated a 3-luminance level improvement. Autosomal dominant-*NR2E3* patients had no improvement
Kumaran *et al*, 2020^23^	19 patients with *RPE65*-related retinal dystrophy	Vision-guided mobility assessment	VA; CS; VF; FST; Impact of Vision Impairment Questionnaire	Completion time; error number; walking speed; PPWS	Repeatability—Large repeatability coefficient of 1.10 m/s.Content validity—Mean retinal sensitivity (p=0.022) and total hill of vision (p=0.022) predicted walking speed with significance. No correlation between walking speed and VA (p=0.340) or CS (p=0.433)Criterion validity—Walking speed approached significance (p=0.052) and was positively associated with affected subjects’ perceived difficulties with mobility
Pierce *et al*, 2024;^66^ Pierce *et al*, 2024^67^	26 patients with *CEP290*-associated retinal dystrophy	Ora-VNC (Visual Navigation Challenge)		Navigation time; Composite score	Content validity—Composite score was correlated with BCVA, white light FST and red light FST in both eyes, and blue light FST in the better eye (p<0.05).Construct validity—Nine participants (64%) showed a meaningful improvement from baseline.Repeatability—Mean test–retest variability from baseline to retest in the worse eye was 0.6 for VNC composite score (95% CI = −0.1 to 1.3).Sensitivity to change—Mean change from baseline to 12 months test in the worse eye was −0.1 (-1.2 to 1.0).
Virtual reality O&M					
Authie *et al*, 2023^29^	30 patients with retinitis pigmentosa	MObility Standardised Test (MOST)	VA; CS; VF; Dark adaptation	Trial duration; Number of collisions; Number of steps and flags touched; Entries in the dead end; Course redirections	Construct validity—Demonstrates discrimination between patients and controls (accuracy larger than 95% in all conditions) and between early and late stages of the disease (mean accuracy of 82.3%).Content validity—Average performance score strongly correlated with VA, CS and VF.Reliability—Highly reproducible (intraclass correlation coefficient >0.98) and reliable (VR and real-life correlation r=0.98).
Aleman *et al*, 2021;^27^ Bennett *et al*, 2023^28^	29 patients with choroideremia, *RPE65-*associated Leber’s congenital amaurosis, *EYS, CNGB1, NR2E3, RPGR, CRKL, PRPH2, USH2A, PRPF31-*associated retinitis pigmentosa	Virtual reality orientation and mobility	VF; FST; VA	Speed; Accuracy (obstacle identification, departures from the path, direction of movement, collisions and whether the subject missed any arrows or repeated them)	Content validity—Better performance in patients with better VA and larger VF extents.Construct validity—Significant difference in the time to complete obstacle testing between patients and controls (p=0.0027). Controls identified approximately 50% of the obstacles at the dimmest course luminance. All but two patients were able to complete the test, although they required higher luminance levels (by >2 log units) to identify 50% of the obstacles.Repeatability—Small improvement in object detection on the second test leading to positive test–retest differences. Greater test–retest values at the dimmest obstacle course luminance level suggest a minor learning effect.
Facial recognition					
Hirji *et al*, 2020; Hirji *et al*, 2021	72 patients with primary open angle glaucoma with glaucomatous macular damage	The Cambridge Face Memory test	VF; CS	Percentage of correctly identified faces	Content validity—Significant correlation between facial recognition and VF mean deviation (p<0.0001)
Observer-rated performance tests					
Azoulay-Sebban *et al*, 2020;^21^ Lombardi *et al*, 2018	32 patients with glaucoma	Homelab at StreetLab	VA; CS; VF; NEI VFQ-25	Path travel time; Mobility incidents; Movement onset; movement initiation time and duration; Localisation of people time; Face orientation recognition time	Construct validity—No significant difference in path travel time between patients and controls. Number of mobility incidents was higher in the advanced glaucoma group than in the other two groups (p=0.0126 and 0.0281, for controls and early glaucoma respectively).Content validity—Integrated binocular field and VF demonstrated significant correlation with test outcomes. Overall movement duration for small objects in reaching and grasping tasks was significantly longer in patients with glaucoma compared with controls. Mobility incidents and the reaching and grasping task parameters were not significantly correlated with quality-of-life questionnaire scores.
Visual search					
Higgins *et al*, 2020^49^	38 patients with non-neovascular age-related macular degeneration	Computer-based assessment (Visual search task and simulated dynamic driving scene)	VA; CS; MP; EuroQol-5D questionnaire	Total correct responses; Median response time	Construct validity—Slower performance in visual search tasks associated with more severe disease. No significant difference between groups for total correct responses (p=0.342). Significant difference in median response time between the groups (p=0.007). Early and intermediate group’s median response time was not significantly slower than the controls.Content validity—Response time was associated with measures of VA and CS.
Kartha *et al*, 2023	37 patients with ultra-low vision	Virtual reality visual performance test	Berkeley Rudimentary Vision Test	Item measure; Person measure	Content validity—Negative correlation between patients with poorer visual acuity having lower person measures (p=0.002, r^2^=0.2, mean absolute error=0.43).Construct validity—Items measures ranged between −1.09 to 0.39 in relative d′ units. Person measures ranged between −0.74 and 2.2 relative d’ units.
Zhang *et al*, 2022;^36^ Manley *et al*, 2022^37^	63 patients with cerebral visual impairment	Virtual toybox and virtual hallway		Success rate; Reaction time; Gaze error; Visual search area; Off-screen per cent (an index of task compliance	Construct validity—For the virtual toybox task, mean success rate for patients was significantly lower compared with controls (p<0.001). Significant difference with respect to mean reaction time with patients taking longer to find the target compared with controls (p<0.001). For the virtual hallway task, the mean success rate for patients was significantly lower compared with controls (p<0.001). Mean reaction time was significantly greater in patients compared with controls (p<0.001).
Driving simulators					
Adrian *et al*, 2022^22^	14 patients with glaucoma	Fixed base driving simulator at StreetLab		Reaction times; Longitudinal regulation; lateral control; eye and head movements; Fixation duration and number per second; Fixation duration; horizontal and vertical gaze direction; head yaw	Construct validity—Compared with controls, patients demonstrated a longer mean duration of lateral excursions (p=0.045), and more lane excursions in a wide left curve (p=0.045). Patients demonstrated a larger SD of horizontal gaze (p=0.034). No significant difference was established for the other measured outcomes.
Lee *et al*, 2019	31 patients with glaucoma	DriveSafe (slide recognition test)	VA; VF; CS; UFOV test	Total number of correctly identified road user features (DriveSafe score); number of fixation points; average fixation duration; average saccade amplitude; horizontal and vertical search variance	Construct validity—Patients had significantly worse DriveSafe scores (p=0.03), fixated on road users for shorter durations (p<0.001), exhibited smaller saccades (p=0.02), reduced fixation duration and saccadic amplitudes compared with controls (p<0.001 and p=0.02). No other significant group differences were found.Content validity—Significant relationship between clinical measures and DriveSafe scores: UFoV 2 (p=0.005), worse‐eye VF mean deviation (p=0.003), CS (p=0.03) and UFoV 3 (p=0.05).

Where a genetic mutation was reported, this has been included in italics. If a form of validation evidence (eg, construct validity) is absent from the table, it was not reported in the original article.

* Indicates a conference abstract.

BCVA, best corrected visual acuity; CS, contrast sensitivity; FLORA, functional low‐vision observer rated assessment; FST, full-field stimulus testing; MP, microperimetry; NEI VFQ-25, National Eye Institute Visual Function Questionnaire-25; POAG, primary open angle glaucoma; PPWS, percentage preferred walking speed; PWS, preferred walking speed; UFOV, useful-field-of-view; VA, visual acuity; VF, visual field; VR, virtual reality.

A clinical reference standard was identified in 29 out of the 45 functional vision tests. Overall, 37 out of 45 functional vision tests reported evidence of statistical validation, but these were of varying robustness. To date, 7 functional vision tests have been used as outcome measures in 10 separate clinical trials for retinal disease as outlined in table 3.

**Table 3:** Functional vision tests used as clinical trial outcome measures

Name of functional vision test	Disease population	ClinicalTrials.gov identifier	Type of outcome measure
Multi Luminance Mobility Test (MLMT)	*RPE65*-related Leber’s congenital amaurosis	NCT00999609	Primary
	*NR2E3 and RHO*-related retinitis pigmentosa	NCT05203939	Efficacy
The Functional Low-Vision Observer Rated Assessment (FLORA for Argus II prosthesis)	End-stage retinitis pigmentosa	NCT02303288; NCT03406416	Primary; Secondary
Low Luminance Mobility Testing (LLMT)	Retinitis pigmentosa	NCT03073733	Secondary
Visual Navigation Challenge (Ora-VNC)	*CEP290*-related Leber’s congenital amaurosis	NCT03140969; NCT03872479	Secondary
Multi-Luminance Y-Mobility Test (MLYMT)	Retinitis pigmentosa	NCT04945772	Secondary
Vision-guided mobility assessment	*RPE65*-related retinal dystrophy	NCT02781480	Secondary
Orientation and mobility for Argus II prosthesis	End-stage retinitis pigmentosa	NCT00407602	Secondary

### Orientation and mobility tests

The most common format of functional vision test was obstacle course, assessing orientation and mobility. Performance on obstacle courses was generally assessed by speed and accuracy, which were often combined to produce an overall score. Metrics of speed include preferred walking speed, percentage of preferred walking speed and course completion time. Accuracy metrics include error number, number of collisions or incidents or path departure. One study provided more detailed metrics on trajectory analyses and walking initiation time aided by measurement tools such as motion capture systems and inertial sensors.^15^ Some tests involved videotaped performances which were sent to reading centres for grading to reduce the risk of grader bias.^16^

Courses ranged in size from 2.1×3.6 m to 68×1.3 m and were located in purpose-built facilities, hospitals and real indoor rooms (eg, a cafeteria). All tests identified in this review were performed indoors, although outdoor mobility tests have been described in the literature.^17 18^ Some tests were performed under multiple luminance levels, ranging from 0.2 to 500 lux, tested in stages to be sensitive to different levels of nyctalopia. No orientation and mobility test exposed patients to acute changes in illumination to test rapid light or dark adaptation, a common difficulty reported in retinitis pigmentosa, perhaps due to safety concerns. Better designed obstacle courses incorporated changes in floor elevation to assess depth perception. If featured in the course, obstacles were commonly made of cardboard or foam and were suspended at various heights. Some tests reported the Weber contrast values and chromaticity coordinates of the obstacles.

Orientation and mobility tests were predominately used on patients with rod-cone dystrophy or glaucoma. As such, the test is suitable for patients with low vision and defects of peripheral vision. The Multi Luminance Mobility Test (MLMT) was used as a primary outcome measure in the landmark clinical trial of voretigene neparvovec (Luxturna) for *RPE65*-related Leber’s congenital amaurosis, the first approved gene therapy in ophthalmology.^19^ The MLMT adopts a binary instead of a continuous scoring system, is performed under seven different luminance levels and demonstrates ceiling effects.^20^ The low luminance conditions allowed the test to demonstrate sensitivity to changes in disease state; *RPE65* is an enzyme which facilitates dark adaptation of viable rod photoreceptors. It follows that a drug capable of rescuing the function of defective *RPE65* would result in enhanced scotopic vision.^19^ The success of the MLMT has subsequently inspired the development of several commercial, academic and dedicated facilities offering functional vision testing, to include Streetlab and Ora.^1521^,^24^ It should, however, be noted that MLMT is primarily an assessment of scotopic vision augmented by dark adaptation of rods and not necessarily the best method to assess cone function.

### Applications of virtual reality technology

VR can overcome many limitations of orientation and mobility tests. VR may absolve the need for a physical, homogeneously lit room while still maintaining a degree of realism.^25^ As such, it is more accessible for use in multicentre clinical trials and can overcome the scaling challenges of physical obstacle courses. However, VR-related motion sickness has been reported, and as a result, patients may still be instructed to walk in physical space to avoid this.^26^ Commonly used VR headsets include the HTC Vive Pro Eye, Fove 0 and Oculus Rift, which are consumer devices commercially available at a relatively low cost. Proprietary, custom-made software was used on this hardware. Some studies included trackers mounted to patients’ head, hands and feet to generate kinematic data.^27 28^ The technical specifications of VR devices were as follows: display screens were LED (Light Emitting Diode) or AMOLED (Active-Matrix Organic Light Emitting Diode), panel sizes ranged from 18.5″ to 80″, resolution ranged from 1280 × 1440 to 4K and the horizontal field of view ranged from 89 to 150 degrees. If reported, the display refresh rate was 90 Hz. VR tests were conducted binocularly, although recent iterations enable monocular testing.^28 29^

### Visual search tests

Visual search tasks relate to several domains of functional vision including social interaction, reading, driving and mobility, and have been used to assess patients with various forms of visual impairment.^30 31^ Visual search may be performed binocularly in front of a display monitor with free head movements or using VR headsets with in-built eye-tracking. Display screen sizes generally range from 17″ to 27″, although a hemispheric, panoramic screen covering 180 degrees of horizontal visual field has been reported.^32^ Eye tracking devices included the Tobii EyeX, Tobii 4C, Tobii Pro X3-120, Tobii AB (Tobii technology, Stockholm, Sweden), HTC Vive trackers (HTC, New Taipei, Taiwan), Oculus Quest Pro (Meta, Burlingame, California, USA) and the Eyelink II system, Eyelink 1000 system (SR Research, Ontario, Canada). Proprietary, custom-made software was used on this hardware. Task performance metrics were search time and correct responses.

Visual scenes included geometric shapes hidden in a computer-generated room and everyday objects hidden in photographs of real-world scenes. Psychophysical targets such as optotypes or geometric shapes are not intuitively reflective of real life, and studies have shown that a Landolt C search task, compared with object identification in a real photograph, did not differentiate patients from visually healthy controls.^33^ All scenes found in visual search tasks were two-dimensional and static, and therefore not reflective of dynamic scenes of the real world. The realism and context provided by real world scenes is important as the role of global features and semantic guidance in object search has been well evidenced to influence visual behaviour.^34 35^ Early iterations of visual search tests in simulated realistic scenes have demonstrated discriminative ability, even in paediatric patients.^36 37^ One portable tablet-based visual search test was able to discriminate patients with severe diabetic macular oedema from an established normative database.^38^

### Driving simulator tests

Driving simulator tests have previously been used to evaluate safety, for example, in glaucoma and in the development of new multifocal intraocular lenses, but not treatment effectiveness in clinical trials.^39 40^ Driving simulator tests have been described in many forms. Moving base driving simulators exist that benefit from a realistic car body and wide-field scene projection but lack the accessibility of other portable simulators.^41^ Desktop-based driving simulators are low fidelity tests, and the lack of real-world consequences from patient error has been reported to influence behaviour by overstating true driving performance.^39^ The artificial driving scenes in these desktop-based simulators can also cause the patient to subtend a smaller visual angle compared with real life, which inadvertently affects the amplitude of saccadic eye movements—a common measure of performance in driving simulator tests.

### Observer-rated visual performance tests

Observer-rated visual performance tests are simulated activities of daily living performed in a controlled environment and assessed by an observer. These tests have been shown to correlate with similar tasks performed at home.^42^ Tested activities include dialling a phone number, reading in reduced illumination or opening a lock with a key. The original Assessment of Function Related to Vision was limited by ceiling effects and was superseded by the Assessment of Disability Related to Vision. The Compressed Assessment of Ability Related to Vision is a compressed version of this test requiring only 14 min to complete. In 2014, the Functional Low-Vision Observer Rated Assessment was developed as an untimed, home-based test for ultra-low vision patients in the context of a clinical trial for the Argus II retinal prosthesis; a validation study is ongoing.^43^ A validation study for the more recently developed Instrumental Activities of Daily Living Tools in Very-Low Vision underscores the tests’ potential as an outcome measure in vision restoration trials. It was developed using a Delphi consensus procedure, with input from occupational therapists and low-vision experts, maintaining high levels of content validity.^44^ Novel observer-rated performance tests are in development with good repeatability and monocular testing.^45^ Limitations of potential observer bias were reported, although newer test iterations have incorporated automated scoring methods using sensors attached to objects to detect object displacement.^46 47^ The tests were also subject to floor and ceiling effects^48^ and could place infeasible cognitive and motor demands on patients in line with the activities assessed, limiting their use to a select subset of suitable patients.

### Facial recognition tests

The Cambridge Face Memory Test is a validated, computer-based, alternative forced choice task where a target face must be distinguished from two additional unfamiliar faces. The test is freely available online, performed binocularly and has an established normative reference score. The test demonstrates variable discriminative ability when applied to different disease cohorts. In patients with dry age-related macular degeneration (AMD), the test was not found to be sensitive to early or intermediate stages of dry AMD but was able to discriminate individuals with features of late-stage disease such as geographical atrophy.^49^ Moreover, one study showed no significant correlation between facial discrimination performance and severity of diabetic macular oedema.^38^ Co-occurring psychiatric illness, neurological damage or neurodevelopmental disorders such as autism affect facial recognition,^50^ and facial recognition tests are used cautiously in these populations.

## Discussion

A functional vision test has been used as a primary outcome measure in a landmark gene therapy clinical trial in ophthalmology. This has set the stage for the development of more unconventional assessments of vision which will be evaluated here.

### Existing functional vision tests in ophthalmology

Orientation and mobility tests were originally used in early clinical trials of retinal prosthesis implants in blind or ultra-low vision patients.^51^,^53^ They were favoured as these patients often had remnants of useful vision and light perception that were not captured in standard clinical tests of visual function. As such, these functional tests have relevance in end-stage disease than in early-stage disease where structural changes remain sensitive markers of clinical progression.^54^ They are useful in measuring low luminance mobility and peripheral vision loss, although individuals with localised degeneration may employ head and eye movements to project the visual environment onto islands of functioning retina. In a study with patients with choroideremia, no deficit in MLMT performance was observed due to preserved macular function even in the presence of advanced peripheral disease.^16^

Orientation and mobility tests are constrained by several limitations, and performance scores can be marred by many sources of error. First, the tests are inherently influenced by patients’ confidence and psychological state. For example, a distinguishing feature of orientation and mobility tests is that an error committed results in an immediate physical response, such as colliding with an obstacle or wall. How individuals negotiate these physical responses varies widely, in terms of risk management or aversion. Furthermore, if patients are aware of being observed or recorded, then the results may be additionally confounded by the Hawthorne effect. The time taken to complete the course is likely influenced by patient confidence, which may improve if a patient is aware that they have received a potentially sight-saving treatment, thereby conferring a placebo effect. Performance scores may also be confounded by a learning effect, and repeated testing is necessary to overcome this, which can prove laborious for patients—if patients are instructed to repeatedly walk as fast as possible in multiple course runs to determine maximum performance speed, they may be limited by physical stamina rather than their vision.

Practically, the resources required to develop, conduct and maintain these tests limit their scalability and may preclude their continued use in multicentre clinical trials. Several orientation and mobility VR tests have been described that offer easy manipulation of the digital visual environment and potentially unlimited course configurations. These tests provide greater optionality in assessing a range of diseases and control of experimental conditions, therefore improving test reproducibility. The automated scoring performance in VR can also reduce assessor bias. Moreover, VR can make an orientation and mobility test into a game by introducing interactive scoring. For example, tests exist that instruct patients to ‘tag’ obstacles with a controller.^28^ However, certain limitations arise from the use of VR. The physical VR headset detaches the user from reality and introduces a degree of abstraction to a task. Discrepancies in resolution between the retina and a VR display screen can affect true perception, particularly if the pixel density and resolution are considerably below human acuity.^55^ VR tests remain in their infancy and require validation in relevant patient populations to ascertain their usability as outcome measures.

VR has also been applied to visual search tests which have demonstrated discriminative ability, even in paediatric patients.^36 37^ The increased accessibility of eye tracking technology as consumer devices, evidenced by the 2024 release of the Apple Vision Pro, assures further development of VR and visual search tests. An avenue of future development may be wearable technologies that can monitor real-time visual search in daily life over extended periods of time. A similar application is the European Medicines Agency (EMA) approved endpoint of wearable sensors that quantify movement in muscular dystrophy trials.^56^

Driving simulator tests have been described in several formats, although if patients have been banned from driving due to deteriorating vision, then the psychological impact of being subjected to a driving test should be considered. Not all patients, particularly those with early onset inherited retinal diseases, ever learn to drive, limiting the accessibility of the test.

### Inherited retinal diseases: a use case for functional vision tests

Well-designed tests of functional vision relate closely to the prevailing symptoms throughout the natural history of an ophthalmological disease. The symptoms of the disease guide test development to ensure that highly relevant concepts of interest are assessed, and that outcomes remain patient-relevant and pertinent to quality of life. Development and validation are challenging in diseases with variable phenotypes or low prevalence, both exhibited within inherited retinal diseases which collectively represent the leading cause of blindness among working age adults in England and Wales.^57^ Pathogenic mutations in over 280 genes have been identified as causing inherited retinal disease; each mutation is associated with its own phenotypic characteristics and so patient symptoms can be highly nuanced.^58^ Selected outcome measures will depend on the underlying disease mechanism and whether a gene-specific or gene-agnostic therapy is developed. The growth of research and development into therapies for these inherited retinal diseases calls for agile innovation in clinical trial outcomes measures to facilitate the arrival of novel gene therapies to market.

Tests that are selected as clinical trial outcome measures should also relate to the region of therapy delivery. For example, in rod-dominated photoreceptor degeneration, the main symptom may be reduced peripheral vision, but if a drug is administered to rescue remaining photoreceptors at the macula, it is logical to preclude the use of a mobility test that may be insensitive to ultimately measure therapy efficacy. This emphasises the importance of judiciously selecting appropriate and effective outcome measures. Additionally, functional vision tests that are performed binocularly have limited utility in clinical trials featuring monocular interventions, particularly where therapy is delivered to the worse seeing eye—as is common practice—as the better seeing eye tends to predict visual functional ability.^59^ Ideally, both monocular and binocular assessments should be performed. Assessments of binocular function can provide understanding of overall function, leading to interpretations of quality of life and subsequent health economic analyses.

Several inherited retinal diseases are syndromic with systemic abnormalities that may additionally impair a patient’s ability to perform a functional vision test, for reasons other than reduced vision due to retinal degeneration. An example of this is in Joubert’s syndrome, whereby mutations in *CEP290* concurrently cause Leber’s congenital amaurosis and psychomotor delay with cerebellar malformations, among other ciliopathy-associated abnormalities.^60^ Performing a functional vision test in these patients with cognitive and physical impairment would be unreliable in measuring changes in retinal function, and it may be difficult to isolate the true measurement of retinal disease due to the confounding effect of systemic abnormalities.

### Challenges in the paediatric validation of functional vision tests

There is a dearth of validated functional vision tests for use in paediatric patients. This is of particular relevance if novel therapies, which are proven to be efficacious in adults, are offered to patients at an earlier age, and in the case of diseases which typically have an early onset of presentation. Examples include Luxturna for *RPE65*-LCA, which used the MLMT in a trial involving adult patients, but for which treatment may be initiated in younger patients as index presentations are frequently early in life. Tests should be optimised for use in children with appropriate modifications to enable clinical trials and post-trial monitoring to capture the benefit conferred by new treatments. Few functional vision tests identified in this review have been used in children.^1523 27 28 36 37 61^,^68^

### Validation of novel functional vision tests

Treatments such as visual prostheses, stem cell transplantation, gene augmentation and editing therapies, antisense oligonucleotide therapy and optogenetic therapies are being developed at pace for previously untreatable ocular conditions.^69^ Progress in the development of these treatments requires validated outcomes. The paucity of validation in functional vision tests is evidenced in table 2 and S2online supplemental table S2. Few articles reported a full description of test methodology to allow replication, and validation evidence was either absent or fragmented. The absence of an established gold standard test for the measurement of functional vision meant no studies were found to report concurrent validity. Clinically adjudicated reference standards to validate novel tests have been reported in other fields of medicine such as infectious disease diagnostics, and may be useful in the absence of an existing gold standard test.^70^

The functional vision tests in this review correlate with clinical measures of visual function to varying degrees of significance and construct validity. The appropriateness of this correlation may be questioned, as functional vision tests measure a distinct aspect of vision rather than acting as surrogate indicators of visual function, raising the issue of whether full validation is required in all cases of test development. It can be said that drawing on the experience of clinicians and patients’ perspectives should provide more weight in determining whether test measurements provide useful and clinically meaningful information.

Most current clinical trials adopt a monocular study design to benefit from the contralateral eye as a control, but the need for standardised, precise and reliable outcome measures will become critical once treatments are delivered bilaterally.^71^ Standardised validation of functional vision tests can improve evidence synthesis, the inferential quality of results and enhance comparability of data between clinical trials with treatments for the same disease. It is reasonable to suggest that functional vision tests should still be validated against standard clinical measures of visual function, but the strength of its validation, or lack thereof, should not solely dictate inclusion as an outcome measure in clinical trials.

In the 1990s, the increase in visual prosthesis development for vision restoration trials led to a greater need for clinically meaningful endpoints. The various centres that developed visual prostheses used different efficacy measurements, making cross-comparison challenging. This led to the International Harmonization of Outcomes and Vision Endpoints in Vision Restoration Trials taskforce, where experts from around the world collaboratively formed guidance to measure visual function in vision restoration clinical trials.^72^ Most functional vision tests found in this review have been applied to inherited retinal diseases, as shown in table 3, yet there is currently no such directive for inherited retinal disease. Novel clinical trial outcome measures would benefit from being guided by consensus-building to retain standardisation. Stakeholders involved in such consensus-building should include patients, advocacy groups, clinical trial sponsors, disease experts, regulatory agencies and experts in the functional vision construct being measured.

### Limitations

The limitations of this review and directions of future research should be considered. A scoping review was selected because of the heterogeneity of the articles identified in the literature search, and it can serve as a foundation for a systematic review or meta-analysis. Test validation in the included studies was reported with varying levels of detail, and as such, in-depth statistical analysis of validation data was not undertaken. Incomplete or insufficiently reported descriptions of tests and data limited the scope of the analysis in some cases. This review aimed to address these limitations by critically evaluating their implications and providing evidence-based recommendations to guide future reporting practices.

Functional vision tests are in development globally, and the regional cultural differences in activities of daily living were not explored in this review, nor were the sources of funding for centres developing functional vision tests. Furthermore, given that functional vision tests assess aspects of higher-order visual processing,^3^ exploring correlations of functional vision performance scores with primary visual cortex activity may also be an avenue for future research.^37^

## Conclusion

Functional vision tests can facilitate research into future novel ophthalmological treatments that prioritise patients in terms of how clinical benefit is defined. The principal barriers to the uptake of these tests are lack of accessibility, low quality validation and that many tests remain early in their development stage. This review captures the current landscape of functional vision tests and serves as a reference for investigators and regulatory bodies to evaluate the suitability of these tests for ophthalmic clinical trials.

## Supplementary material

10.1136/bmjopen-2024-097970 online supplemental file 1

## Data Availability

All data relevant to the study are included in the article or uploaded as supplementary information.
