# Supplementary material for: Functional vision tests as clinical trial outcome measures in ophthalmology: a scoping review
Source: BMJ Open. 2025 May 27;15(5):e097970. doi: 10.1136/bmjopen-2024-097970 (PMC12121612; doi:10.1136/bmjopen-2024-097970)
Supplement: online supplemental file 1 [file bmjopen-15-5-s001.docx]

**Supplementary Material**

Table S1. Full Boolean search strategy divided into two concepts: functional vision and eye disease

| Search strategy performed in MEDLINE and Embase (via Ovid) on 1^st^ August 2024 |
| --- |
| Functional vision.ti,ab.  Functional ability.ti,ab.  Functional disability.ti,ab.  Functional impairment.ti,ab.  Performance based.ti,ab.  Real world vision.ti,ab.  Real world task.ti,ab.  Daily living task*.ti,ab.  Mobility.ti,ab.  Vis* task.ti,ab.  Visual search.ti,ab.  Eye-Tracking Technology/  Fac* recognition.ti,ab.  1 or 2 or 3 or 4 or 5 or 6 or 7 or 8 or 9 or 10 or 11 or 12 or 13  Eye Diseases/  Visual* impair*.ti,ab.  Vision impaired.ti,ab.  Glaucoma/  Inherited retinal disease.ti,ab.  Achromatopsia.ti,ab.  Choroideremia.ti,ab.  Stargardt Disease/  Usher Syndromes/  Leber Congenital Amaurosis/  Optic Atrophy, Hereditary, Leber/  Retinitis Pigmentosa/  Macular Degeneration/  15 or 16 or 17 or 18 or 19 or 20 or 21 or 22 or 23 or 24 or 25 or 26 or 27  14 and 28  limit 29 to (english language and yr="2003 -Current") |

Table S2. Patient population, reference standard, test outcomes, and repeatability and validity data of all included studies featuring a functional vision test

| Citation | Patient population | Functional vision test | Reference standard(s) | Test outcome(s) | Reported repeatability and validity data |
| --- | --- | --- | --- | --- | --- |
| Orientation and mobility (O&M) | | | | | |
| Roman et al., 2022 | 10 patients with *GUCY2D-* and *CEP290-* associated Leber’s congenital amaurosis | **Mobility test for rod-mediated vision** | VA; FST | Navigation success over a fixed number of trials; Travel duration | **Content validity** - Mobility demonstrated a linear relationship with FST. No correlation between VA and mobility  **Construct validity** - No significant difference between controls and patients in suprathreshold transit time (p=0.63). At threshold and dimmer luminance levels, transit times increased for both patients and normal subjects. |
| Sahel et al., 2021  Bertaud et al., 2021 | 25 patients with retinitis pigmentosa and *RPE65-*associated Leber’s congenital amaurosis  22 patients with glaucoma | **StreetLab mobility course** | VA; VF; CS; Dark adaptation | Course completion time; PWS; PPWS; Number of collisions; Walking initiation time; trajectory analyses/segments; Distance travelled | **Construct validity** – Patients performed worse than controls for PWS, PPWS, number of collisions and walking initiation time under both low and high illumination.  **Construct validity** – No difference in mobility performance between patients and controls under photopic luminance. Under glare conditions, PWS and PPWS were significantly lower in patients than controls (p=0.049 and p=0.038 respectively). Mobility time was significantly longer in patients than controls (p=0.046). Distance travelled, mobility incidents, and trajectory segmentations not significantly different between patients and controls. |
| Chung et al., 2018; Maguire et al., 2019  Maguire et al., 2021  Lam et al., 2024* | 19 patients with *RPE65-*associated Leber’s congenital amaurosis  19 patients with *RPE65-*associated Leber’s congenital amaurosis  18 patients with *NR2E3 and RHO-*associated retinitis pigmentosa | **Multi-Luminance Mobility Test (MLMT)** | VA; VF; FST (white light) | MLMT binocular change score (number of collisions and time to navigate course)  MLMT monocular change score | **Content validity** - Variable correlation of accuracy score with quality-of-life questionnaire (r=−0.54 to −0.7). Correlation of mean accuracy score with VA ranged from 0.75 to 0.86. Correlation between mean accuracy score and total degrees of visual field ranged from −0.37 to −0.53.  **Construct validity** - Able to distinguish controls from patients.  **Repeatability-** High inter-grader agreement for scoring (Cohen’s kappa=97.9%). High concordance between scores at baseline visits ranging from 86% to 98%.  **Sensitivity to change** - Over 1-year observation period controls had an MLMT change score of 0, representing no change and 20 patients had an MLMT change score of 0. Few patients had an MLMT change score of −1 or −2 (i.e. a worsening).  **Construct validity –** 6 out of 7 *RHO* patients had stable or improved MLMT scores, including 2 patients that demonstrated a 3-luminance level improvement. Autosomal dominant-*NR2E3* patients had no improvement |
| Kammer et al., 2021* | 20 patients with retinitis pigmentosa | **Low Luminance Mobility Test (LLMT)** | VA; CS; VF; VA LV VFQ-48 | Critical Illumination Level; Maximum Step Speed score | **Content validity -** All visual function measures significantly related to Critical Illumination Level in a multiple regression model, R² =0.75 (p=0.004)  **Construct validity** - Able to distinguish controls from patients.  **Repeatability** - No change in Critical Illumination Level between test sessions for 75% of patients. Inter-rater and intra-rater grading biases close to zero and no significant differences between graders (p>0.05). |
| Xu et al., 2021 | 5 patients with retinitis pigmentosa | **Orientation and mobility test (256 Channel Intelligent Micro Implant Eye implant)** |  | Effort; Average completion time | Not reported |
| Boyer et al., 2023* | 27 patients with advanced retinitis pigmentosa | **Multi-Luminance Y-Mobility Test (MLYMT)** |  |  | Not reported |
| Kumaran et al., 2020 | 19 patients with *RPE65*-related retinal dystrophy | **Vision-guided mobility assessment** | VA; CS; VF; FST; Impact of Vision Impairment Questionnaire | Completion time; error number; walking speed; PPWS | **Repeatability** – Large repeatability coefficient of 1.10 m/s.  **Content validity -** Mean retinal sensitivity (p=0.022) and total hill of vision (p=0.022) predicted walking speed with significance. No correlation between walking speed and VA (p=0.340) or CS (p=0.433)  **Criterion validity** - Walking speed approached significance (p=0.052) and was positively associated with affected subjects’ perceived difficulties with mobility |
| Jacobson et al., 2017 | 22 patients with *CEP290-*associated Leber’s congenital amaurosis | **Mobility performance task** | FST | Number of patient incidents (obstacles/wall bumps or reorientations) | **Content validity** – Correlation between mobility score and VA (p =0.002). |
| Alshaghthrah et al., 2014; Al Saqr et al., 2017 | 20 patients with retinitis pigmentosa | **Portable mobility course** | VA; CS | PPWS; Collision score | **Content validity** - Significant correlation between VA and collision incidences (p=0.03). No significant correlation between CS and mobility scores (p > 0.05).  **Repeatability** - PPWS scores not significantly different (p>0.05) on repeat testing. Collision incidences significantly lower at the second visit (p=0.012). Agreement of collision incidences between the two visits suggestive of no learning effect. |
| Shapiro et al., 2017*;  Pierce et al., 2024; Pierce et al., 2024  Russell et al., 2022 | Inherited retinal disease  26 patients with *CEP290*-associated retinal dystrophy  11 patients with *CEP290*-associated Leber congenital amaurosis | **Ora-VNC (Visual Navigation Challenge)** |  | Navigation time; Composite score | **Construct validity** - Navigation times for controls, mild and severe retinitis pigmentosa were significantly different across all light levels (p<0.05) and between groups (p < 0.05).  **Content validity** – Composite score was correlated with BCVA, white light FST and red light FST in both eyes, and blue light FST in the better eye (p < 0.05).  **Construct validity** – Nine participants (64%) showed a meaningful improvement from baseline.  **Repeatability –** Mean test-retest variability from baseline to retest in the worse eye was 0.6 for VNC composite score (95% confidence interval = −0.1, 1.3).  **Sensitivity to change –** Mean change from baseline to 12 months test in the worse eye was -0.1 (-1.2, 1.0).  **Construct validity** - Mean (±standard deviation) improvement in composite score was +2.50±3.118 in treated eyes compared to +1.75±2.383 in untreated eyes (p=0.10). A greater improvement in the composite score from baseline to month 12 was seen in the lower dose group (+4.00±3.114 and +2.67±2.714 for treated and untreated eyes, respectively) compared to the higher dose group (+0.25±1.323 and +0.38±0.750, respectively). |
| Ivanov et al., 2016 | 25 patients with retinitis pigmentosa | **Natural environment walking task with eye tracking** |  | PPWS; Number of obstacle collisions; Eye position variability | **Construct validity** - Average PPWS for controls (92%) was higher than all other patient groups. |
| Ikeda et al., 2015 | 8 patients with retinitis pigmentosa | **Walking test** |  | Number of trial failures; Time taken to reach goal | Not reported |
| Nau et al., 2014 | 36 patients with low vision | **Obstacle course for BrainPort device** |  | PPWS; Percentage of obstacle collisions | Not reported |
| Geruschat et al., 2012 | 8 patients with advanced retinitis pigmentosa | **Orientation and mobility assessment in retinal prosthesis** | VA; VF | Course completion time; Obstacle contacts | **Construct validity** – Significantly increased obstacle contacts between subjects with worse and those with better VA and VF. No significant difference in course completion time |
| Kiser et al., 2008 | 22 patients with age-related macular degeneration | **Mobility obstacle course** |  | Course completion time; Obstacle contacts | Not reported |
| Fuhr et al., 2007 | 44 patients with severe visual impairment | **High density obstacle course** |  | Course completion time; Obstacle contacts | **Construct validity** – Longer course completion time in patients than age matched controls with significant group effect (p<0.0005). Patients made more obstacle contacts than controls. Analyses of mean number of obstacle contacts showed a significant group effect (p=0.001). |
| Velikay Parel et al., 2007 | 10 patients with retinitis pigmentosa, Usher syndrome and optic nerve atrophy | **Mobility assessment** | VA; VF | Average speed; Obstacle contacts | **Content validity** - VA and VF had no significant effect on passing time (p=0.08 and p=0.23 respectively)  **Construct validity** - Average passing times between the groups were significantly different (p=0.03). No significant difference in the average number of contacts between groups (p=0.15) |
| Virtual reality O&M | | | | | |
| Authie et al., 2023 | 30 patients with retinitis pigmentosa | **MObility Standardised Test (MOST)** | VA; CS; VF; Dark adaptation | Trial duration; Number of collisions; Number of steps and flags touched; Entries in the dead end; Course redirections | **Construct validity** - Demonstrates discrimination between patients and controls (accuracy larger than 95% in all conditions) and between early and late stages of the disease (mean accuracy of 82.3%).  **Content validity** - Average performance score strongly correlated with VA, CS and VF.  **Reliability** - Highly reproducible (intraclass correlation coefficient>0.98) and reliable (VR and real-life correlation r=0.98**)** |
| Aleman et al., 2021; Bennett et al., 2023 | 29 patients with choroideremia, *RPE65-*associated Leber’s congenital amaurosis, *EYS-, CNGB1-, NR2E3-, RPGR-, CRKL-, PRPH2-, USH2A-, PRPF31-*associated retinitis pigmentosa | **Virtual reality orientation and mobility** | VF; FST; VA | Speed; Accuracy (obstacle identification, departures from the path, direction of movement, collisions, and whether the subject missed any arrows or repeated them) | **Content validity** – Better performance in patients with better VA and larger VF extents  **Construct validity** – Significant difference in the time to complete obstacle testing between patients and controls (p=0.0027). Controls identified approximately 50% of the obstacles at the dimmest course luminance. All but two patients were able to complete the test, although they required higher luminance levels (by >2 log units) to identify 50% of the obstacles.  **Repeatability –** Small improvement in object detection on the second test leading to positive test-retest differences. Greater test-retest values at the dimmest obstacle course luminance level suggestive of a minor learning effect. |
| Daga et al., 2017 | 31 patients with glaucoma | **Virtual Environment Human Navigation Task (VEHuNT)** | VF | Time to complete task | **Construct validity** - Significant difference on average time to complete task between patients and controls for room A (p=0.001). No significant difference on average time to complete the task between patients and controls for room B (p=0.514). Significant relationship between time to complete the task and visual field loss for room A but not for room B (p=0.001). |
| Facial recognition | | | | | |
| Hirji et al., 2020; Hirji et al., 2021  Glen et al., 2012; Glen et al., 2013  Mazzoli et al., 2019  Taylor et al., 2018 | 72 patients with primary open angle glaucoma with glaucomatous macular damage  54 patients with glaucoma  64 patients with age-related macular degeneration and 48 patients with primary open angle glaucoma  30 patients with non-neovascular age-related macular degeneration | **The Cambridge Face Memory test** | VF; CS | Percentage of correctly identified faces | **Content validity** - Significant correlation between facial recognition and VF mean deviation (p<0.0001)  **Construct validity** - Patients with advanced VF defects identified fewer faces on average than those with early and moderate defects and controls (p<0.05).  **Construct validity** – Test scores were lower in patients compared to controls (p<0.001).  **Construct validity** - Geographic atrophy patients identified significantly fewer faces on average than early and intermediate AMD patients and controls (p=0.04). |
| Observer-rated performance tests | | | | | |
| Delyfer et al., 2021  Karapanos et al., 2021, Petoe et al., 2021  Greenberg et al. 2015  Yoon et al., 2021  Geruschat et al., 2015 | 18 patients with retinitis pigmentosa  4 patients with retinitis pigmentosa  30 patients with retinitis pigmentosa  5 patients with retinitis pigmentosa  26 patients with retinitis pigmentosa | **Functional Low-Vision Observer Rated Assessment (FLORA)** |  | Final impact rating; Task performance score | Not reported |
| Altangerel et al., 2006 | 43 patients with primary open angle glaucoma | **Assessment of Function Related to Vision (AFREV)** | VF; VA; CS | AFREV score | **Content validity** - AFREV scores highly correlated with CS (r = 0.772), binocular VA (r=−0.768), better-eye VA (r =−0.737), worse-eye VA (r =−0.675), and VF scores (r = 0.606) and NEI-VFQ scores (r = 0.70).  **Construct validity** – Distinguishes between mild, moderate and severe binocular VF loss. |
| Kulkarni et al., 2012;  Warrian et al., 2010;  Warrian et al., 2009  Richman et al., 2010, Richman et al., 2010 | 192 patients with glaucoma  91 patients with diabetic retinopathy  112 patients with age-related macular degeneration  192 patients with glaucoma | **Assessment of Disability Related to Vision (ADREV)** | VF  VA; CS; VF; VFQ-25  VA; CS; VF; VFQ-25  VA; CS; VF; Stereopsis | ADREV score | **Content validity** - Highest correlation with the total ADREV score was the integrated VF score (p=-0.49).  **Content validity** – All of the ADREV’s scales were correlated with one or more clinical measures of visual function except the Ambulation test.  **Content validity** – 66% of correlations made between clinical ophthalmic measurements and ADREV scores were significant to P<0.0007. 55% of correlations made between the ADREV and the VFQ total and subscale scores were significant to P< 0.0004.  **Content validity** – ADREV performance was strongly associated with binocular VA (P<0.001) and binocular CS (P<0.001). Monocular and binocular VF results had a weaker correlation with the ability to perform the ADREV tasks (P<0.05). |
| Edwards et al., 2018 | 6 patients with advanced retinitis pigmentosa implanted with Retina Implant Alpha AMS - *USH2A, PDE6B, RPE65, RPGR, CERKL* | **Tabletop object and clock face recognition** |  | No. of correctly location and named items | Not reported |
| Azoulay-Sebban et al., 2020; Lombardi et al., 2018 | 32 patients with glaucoma | **Homelab at StreetLab** | VA; CS; VF; NEI VFQ-25 | Path travel time; Mobility incidents; Movement onset; movement initiation time and duration; Localisation of people time; Face orientation recognition time | **Construct validity** - No significant difference in path travel time between patients and controls. Number of mobility incidents was higher in advanced glaucoma group than in other 2 groups (p=0.0126 and 0.0281, for controls and early glaucoma respectively).  **Content validity** – Integrated binocular field and VF demonstrated significant correlation with test outcomes. Overall movement duration for small objects in reaching and grasping tasks was significantly longer in glaucoma patients compared with controls. Mobility incidents and the reaching and grasping task parameters were not significantly correlated with quality-of-life questionnaire scores. |
| Wei et al., 2012  Sun et al., 2016  Waisbourd et al., 2019 | 9 patients with glaucoma  161 patients with glaucoma  153 patients with glaucoma | **CAARV (Compressed Assessment of Ability Related to Vision)** | VA; CS; VF  VF  VA; CS; VF; VFQ-25 | Total CAARV score | **Content validity** – Strongest correlation was between the central VF cluster and total CAARV score (P<0.001). Central VF cluster in the better eye positively correlated with the majority of CAARV and NEI VFQ-25 subscales.  **Construct validity** – Compared to non-rapid progressors, patients who had rapidly progressing glaucoma presented with lower baseline CAARV scores for reading street signs (p=0.01), facial recognition (p=0.01), and total score (p<0.001). |
| Reighard et al., 2019 | 145 patients with glaucoma | **I-CAARV (Indian - Compressed Assessment of Ability Related to Vision)** | VA; VF; CS; Indian-VFQ | I-CAARV score | **Content validity -** I-CAARV scores and the Indian-VFQ were significantly correlated (P<0.01). Rasch-calibrated scores on the I-CAARV were also significantly correlated with VF MD, presenting VA, best-corrected VA, and CS in both the better-seeing eye (p=0.60, p=-0.51, p=-0.53, p=0.76 respectively) and worse-seeing eye (p=0.48, p=-0.61, p=-0.53, p=0.69).  **Repeatability –** Rasch analysis found that the I-CAARV had moderate reliability (0.74) and measurement precision was fair (person separation 1.67 logits).  Rasch analysis found good **construct validity** (infit range 0.66-1.13; outfit range 0.65-1.21) |
| Peterson et al., 2023* | 36 patients with age-related macular degeneration | **Performance-based activities of daily living task tests (ADLTT)** | VA; CS; MP | Task completion time | **Construct validity –** Longer task completion time in patients than controls for money counting task using worse eye vision and binocular vision (both p<0.001) and on drink making task using monocular worse eye vision (p=0.033).  **Content validity –** Only the money counting task demonstrated moderate to strong correlations with VA, CS, and MP. **Divergent validity** was demonstrated when correlated with race and gender in most ADLTTs except for facial expression task.  **Repeatability** - Moderate to good test-retest reliability for money counting and drink making tasks only using monocular worse eye vision. |
| Ni et al., 2012 | 64 patients with age-related cataract | **Real-Life Vision Test (RLVT)** | VA; CS; Stereopsis; Colour perception; VFQ-25 | Time taken to complete task | **Construct validity –** Controls performed significantly better than patients (P<0.01). Significant difference reported between patients with different cataract severity  **Content validity** - All RLVT subscales remained highly associated with most clinical measures, after controlling for age, years of education, Mini Mental State Examination scores, self-rating depression scores, and reaction time. |
| Finger et al., 2014 | 40 patients with rod-cone dystrophy | **Very Low Vision Instrumental Activities of Daily Living (IADL-VLV)** | VA; VF | Completion and accuracy score | **Content validity –** VA and VF were associated with IADL performance.  **Construct validity –** Patients with worse VA or VF scored lower (p<0.00 and p=0.001 respectively) |
| Visual search | | | | | |
| Higgins et al., 2020  Taylor et al., 2017 | 38 patients with non-neovascular age related macular degeneration  31 patients with dry age-related macular degeneration | **Computer based assessment (Visual search task and simulated dynamic driving scene)** | VA; CS; MP; EuroQol-5D questionnaire  VA; CS | Total correct responses; Median response time  Median search time; Fixation duration; Saccadic amplitude; Saccades per second | **Construct validity** - Slower performance in visual search tasks associated with more severe disease. No significant difference between groups for total correct responses (p=0.342). Significant difference in median response time between the groups (p=0.007). Early and intermediate group’s median response time were not significantly slower than the controls.  **Content validity** - Response time was associated with measures of VA and CS.  **Content validity** – Significant associations between average search time and VA (p<0.001) and CS (p<0.001)  **Construct validity –** 61% of patients exceeded the 90% normative limits for average search time; this was statistically significant (p<0.0001). No differences between groups in fixation duration or saccades per second. Yet saccadic amplitude remained significantly smaller for patients compared to controls (p<0.001). |
| Thibaut et al., 2018 | 21 patients with age related macular degeneration | **Object search in realistic panoramic scenes** |  | Percentage of correct target detection; percentage of false positives; scene views explored; search time | **Construct validity -** No significant differences in performance between patients and age-matched controls. |
| Wan et al., 2020 | 30 patients with age-related cataract | **Visual search and facial recognition task** |  | Fixation count and total duration; total visit duration; Forward and backward saccade count per line; percentage of regressive saccades; percentage of correctly identified faces | **Construct validity** – Significant difference before and after surgery for the percentage of correctly identified objects and faces (p=0.049 and p=0.004 respectively), average search time (p<0.001), fixation count (p<0.001), total fixation duration (p= 0.039) and total visit duration (p=0.008). No significant change was in mean fixation duration.  **Repeatability** - No significant difference between baseline and follow-up assessment (all parameters p<0.05) |
| Kartha et al., 2023 | 37 patients with ultra-low vision | **Virtual reality visual performance test** | Berkeley Rudimentary Vision Test | Item measure; Person measure | **Content validity –** Negative correlation between patients with poorer visual acuity having lower person measures (p=0.002, r^2^=0.2, mean absolute error=0.43).  **Construct validity** – Items measures ranged between −1.09 to 0.39 in relative d′ units. Person measures ranged between -0.74 and 2.2 relative d’ units. |
| Martínez-Almeida et al., 2021 | 33 patients with glaucoma | **Virtual reality system with gaze monitoring** |  | Fixation number and duration; Saccadic amplitude and velocity; Fixation/saccade ratio; Total search and execution time; Number of collisions | **Construct validity** – Significant differences between controls and patients for the static task in terms of number of fixations (p=0.012), mean saccadic velocity (p=0.023 and 0.017), fixations/saccades ratio (p=0.035 and 0.04), and the search and total execution times during visual search exercise (p=0.004 and 0.027, respectively). For the dynamic task, Significant differences were found on average saccades amplitude (p=0.02), average saccades velocity (p=0.03) and the number of collisions (p=0.02). |
| Kurek et al., 2023* | 30 patients with retinitis pigmentosa | **Virtual reality visual search task with natural scenes** | CS | Performance score (encompassing search duration and rate of performance success) | **Construct validity –** Able to discriminate between patients and controls (Accuracy >86%)  **Repeatability** – Good agreement of performance score between sessions (Intraclass correlation coefficient>0.89)  **Content validity** - Correlation with CS was p=0.76. 83% of RP participants indicated that the virtual reality test was representative of their difficulties in daily life. |
| Zhang et al., 2022; Manley et al., 2022 | 63 patients with cerebral visual impairment | **Virtual toybox and virtual hallway** |  | Success rate; Reaction time; Gaze error; Visual search area; Off-screen percent (an index of task compliance | **Construct validity** – For the virtual toybox task, mean success rate for patients was significantly lower compared to controls (p<0.001). Significant difference with respect to mean reaction time with patients taking longer to find the target compared to controls (p < 0.001). For the virtual hallway task, mean success rate for patients was significantly lower compared to controls (p<0.001). Mean reaction time was significantly greater in patients compared to controls (p<0.001) |
| Roux-Sibilon et al., 2018 | 22 patients with glaucoma | **Scene and face recognition** | VF | Participant's response; Reaction time for response | **Construct validity** - Patients demonstrated deficit in both detection and categorization of all low-contrast images compared to controls. |
| Smith et al., 2012 | 40 patients with glaucoma | **Visual search task with eye tracking** | VF; CS | Average number of saccades per second; average saccade amplitude; Average search duration | **Construct validity** - Average rate of saccades by patients was significantly smaller than controls during the visual search task (p=0.02). No difference in average saccade amplitude between the patients and controls (p=0.09).  **Content validity** - Average number of saccades was weakly correlated with CS (p=0.006) and more severe VF defects (p=0.037). |
| Driving simulators | | | | | |
| Adrian et al., 2022 | 14 patients with glaucoma | **Fixed base driving simulator at StreetLab** |  | Reaction times; Longitudinal regulation; lateral control; eye and head movements; Fixation duration and number per second; Fixation duration; horizontal and vertical gaze direction; head yaw | **Construct validity -** Compared to controls, patients demonstrated a longer mean duration of lateral excursions (p=0.045), and more lane excursions in a wide left curve (p=0.045). Patients demonstrated a larger standard deviation of horizontal gaze (p=0.034). No significant difference was established for the other measured outcomes. |
| Kübler et al., 2015 | 6 patients with glaucoma | **Simulated driving test** |  | Driving lane positions; time to line crossing (indicates steering stability); driving speed; head and eye tracking | Not reported |
| Lee et al., 2019 | 31 patients with glaucoma | **DriveSafe (slide recognition test)** | VA; VF; CS; UFOV® test | Total number of correctly identified road user features (DriveSafe score); number of fixations points; average fixation duration; average saccade amplitude; horizontal and vertical search variance | **Construct validity** - Patients had significantly worse DriveSafe scores (p=0.03), fixated on road users for shorter durations (p<0.001), exhibited smaller saccades (p=0.02), reduced fixation duration and saccadic amplitudes compared to controls (p<0.001 and p=0.02). No other significant group differences were found.  **Content validity** - Significant relationship between clinical measures and DriveSafe scores: UFoV 2 (p=0.005), worse‐eye VF mean deviation (p=0.003), CS (p=0.03) and UFoV 3 (p=0.05). |
| Devos et al., 2018 | 17 patients with glaucoma | **Performance based visual field test in a driving simulator** | VF; UFOV® | Total crashes; Speed exceedances; Correct stops at traffic lights; Centre line crossings; Road edge excursions; Complex response time; Target identification accuracy; Number of missed responses; Response time | **Construct validity** - Patients identified fewer VF symbols (p=0.047) and took longer (p=0.048) to detect the VF symbols compared to controls. No significant differences for the other driving performance measures.  **Content validity** - Correlation between performance-based VF test scores and horizontal FOV of the Keystone vision screener and UFOV® divided attention subtest (p=0.02 and p=0.046 respectively).  **Repeatability** – Intraclass correlation ranged between 0.77 for response time and 0.92 for correct responses. |
| Prado-Vega et al., 2013 | 23 patients with glaucoma | **Driving simulator with eye-scanning** | VF | Steering activity; Lane keeping; Longitudinal and lateral distance to obstacle; Collisions | **Construct validity** - No significant difference between patients and controls for lane keeping, obstacle avoidance, and eye-scanning behaviour. Steering activity was significantly higher for patients than for controls.  **Content validity** – No significance correlation between the percentage of depressed IVF points and driving performance measures (p>0.2). |

VA = visual acuity; BCVA = best corrected visual acuity; VF = visual field; CS = contrast sensitivity; MP = microperimetry; FST = Full-field stimulus testing; FLORA = functional low‐vision observer rated assessment; PWS = preferred walking speed; PPWS = percentage preferred walking speed; O&M = orientation and mobility; POAG: primary open angle glaucoma; AMD: age-related macular degeneration; VFQ-25 = Visual Functioning Questionnaire-25; VA LV VFQ-48 = Veterans Affairs Low-Vision Visual Functioning Questionnaire; UFOV = useful-field-of-view. *Indicates a conference abstract. Where a genetic mutation was reported, this has been included in italics. If a form of validation evidence (e.g. construct validity) is absent from table, it was not reported in the original article.
