# Supplementary material for: Functional vision tests as clinical trial outcome measures in ophthalmology: a scoping review
Source: BMJ Open. 2025 May 27;15(5):e097970. doi: 10.1136/bmjopen-2024-097970 (PMC12121612; doi:10.1136/bmjopen-2024-097970)
Supplement: Supplementary file 2 [file bmjopen-2024-097970.reviewer_comments.pdf]

PEER REVIEW HISTORY

BMJ Open publishes all reviews undertaken for accepted manuscripts. Reviewers are asked to complete a checklist review form and are provided with free text boxes to elaborate on their assessment. These free text comments are reproduced below.

ARTICLE DETAILS

Title (Provisional)

Functional vision tests as clinical trial outcome measures in ophthalmology: a scoping review

Authors

Raji, Shabnam; Thirunavukarasu, Arun; Taylor, Laura Jayne; MacLaren, Robert Edward

---

VERSION 1 - REVIEW

**Reviewer**	**1**
**Name**	**Mohamed, S**
**Affiliation**	**Al-Azhar University**
**Date**	**30-Mar-2025**
**COI**	**None**

Good job

**Reviewer**	**2**
**Name**	**Yu, Bo**
**Affiliation**	**Tianjin Medical University Eye Hospital, Glaucoma**
**Date**	**03-Apr-2025**
**COI**	**None**

This review provides an overview of the current state of functional vision tests in ophthalmology and their potential use in clinical trials. It identifies key areas for future development and validation to improve their utility as outcome measures. The review is well-structured and follows established guidelines. Some parts need to be improved.

1. Introduction Part: The initial paragraph defines functional vision. The author then contrasts this with the current standard visual function measures and their limitations. Furthermore, it is highlighted that functional vision is now recognized as a pivotal clinical trial endpoint in neurological diseases. This section should be expanded to include two

additional elements. Firstly, a detailed emphasis on the significance of functional vision tests in ophthalmology clinical trials, particularly in comparison to conventional visual function assessments. Secondly, a comprehensive overview of the prevalent functional vision tests employed in contemporary ophthalmology clinical trials, along with an analysis of their respective constraints.

2. At last, the author should propose suggestions for future research, particularly on how to improve functional vision tests to make them more suitable for ophthalmology clinical trials.

3. Due to the heterogeneity in reported test validation, only a qualitative synthesis was possible. Incomplete data in some studies limited the analysis. This should be mentioned in the limitation part.

**Reviewer**	**3**
**Name**	**Lau, Kian-Chi**
**Affiliation**	**China Medical University Hospital, Department of Acupuncture**
**Date**	**08-Apr-2025**
**COI**	**None**

It's my honour to review your article, which is clinically significant and well-structured.

Following are my question and comment.

Abstract and Results

Abstract: "Of 2,995 articles returned by the search, ……"

Results: "The initial search yielded 2,665 articles."

Which number is correct? 2995 or 2665?

Discussion- Validation of novel functional vision tests

"Treatments such as visual prostheses, stem cell transplantation, gene augmentation and editing therapies, antisense oligonucleotide therapy and optogenetic therapies are being developed at pace for previously untreatable ocular conditions."

References should be added to support this sentence.

VERSION 1 - AUTHOR RESPONSE

Reviewer: 1
Dr. S Mohamed, Al-Azhar University

Comments to the Author:
Good job

Thank you, we appreciate the kind comment from Reviewer 1.

Reviewer: 2
Dr. Bo Yu, Tianjin Medical University Eye Hospital

Comments to the Author:
This review provides an overview of the current state of functional vision tests in ophthalmology and their potential use in clinical trials. It identifies key areas for future development and validation to improve their utility as outcome measures. The review is well-structured and follows established guidelines. Some parts need to be improved.

1. Introduction Part: The initial paragraph defines functional vision. The author then contrasts this with the current standard visual function measures and their limitations. Furthermore, it is highlighted that functional vision is now recognized as a pivotal clinical trial endpoint in neurological diseases. This section should be expanded to include two additional elements. Firstly, a detailed emphasis on the significance of functional vision tests in ophthalmology clinical trials, particularly in comparison to conventional visual function assessments. Secondly, a comprehensive overview of the prevalent functional vision tests employed in contemporary ophthalmology clinical trials, along with an analysis of their respective constraints.
2. At last, the author should propose suggestions for future research, particularly on how to improve functional vision tests to make them more suitable for ophthalmology clinical trials.
3. Due to the heterogeneity in reported test validation, only a qualitative synthesis was possible. Incomplete data in some studies limited the analysis. This should be mentioned in the limitation part.

We appreciate the suggestions made by Reviewer 2.

1 and 2. We did not include this in the introduction as we have explored all points raised by Reviewer 2 in the Discussion. In the Discussion, we evaluate the significance of functional vision tests in ophthalmology clinical trials and exhaustively list the current functional vision tests employed in ophthalmology in Table 1. We also make general recommendations on how to improve future functional vision tests. Due to the sheer heterogeneity of functional vision tests, it is difficult to make specific recommendations and is not appropriate in the context of this scoping review. We did not think it would be appropriate to highlight these points prematurely in the Introduction, as this is the focus of the rest of the review.

3. We have now expanded the limitations section entirely and included the sentence as recommended by Reviewer 2.

Reviewer: 3
Dr. Kian-Chi Lau, China Medical University Hospital, China Medical University College of Chinese Medicine

Comments to the Author:
It’s my honour to review your article, which is clinically significant and well-structured.
Following are my question and comment.

Abstract and Results
Abstract: “Of 2,995 articles returned by the search, ……”
Results: “The initial search yielded 2,665 articles.”
Which number is correct? 2995 or 2665?

Discussion- Validation of novel functional vision tests
“Treatments such as visual prostheses, stem cell transplantation, gene augmentation and editing

therapies, antisense oligonucleotide therapy and optogenetic therapies are being developed at pace for previously untreatable ocular conditions."
References should be added to support this sentence.

We appreciate the kind comments from Reviewer 3 and their suggestions.

The incorrect number of the search articles was an oversight on our part and has now been amended in the abstract to the correct value of 2,665.

We agree that the statement made in the Discussion as highlighted by Reviewer 3 requires a reference and have now included this citation.
